# Population based estimates of non-fatal injuries in the capital of Iran

**DOI:** 10.1186/1471-2458-11-608

**Published:** 2011-07-31

**Authors:** Soheil Saadat, Mostafa Mafi, Mahdi Sharif-Alhoseini

**Affiliations:** 1 Sina Trauma Research Center, Tehran University of Medical Sciences, Tehran, Iran

## Abstract

**Background:**

Fatal injuries are at the top of the injury pyramid; however, non-fatal injuries are quite common and impose huge medical expenses on the population. Relying on hospital data will underestimate the incidence of non-fatal injuries. The aim of this study was to estimate the annual incidence and out of pocket medical expenses of all injuries in urban population of Tehran (the capital city of Iran).

**Methods:**

Using the cluster random sampling approach, a household survey of residents of greater Tehran was performed on April 2008. At randomly selected residential locations, interviewers asked one adult person to report all injuries which have occurred during the past year for all household members, as well as the type of injury, place of occurrence, the activity, cause of accidents resulting in injuries, the amount of out of pocket medical expenses for injury, and whether they referred to hospital.

**Results:**

This study included 2,450 households residing in Tehran during 2007-8. The annual incidence of all injuries was 188.7 (180.7-196.9), significant injuries needing any medical care was 68.8 (63.7-74.2), fractures was 19.3 (16.6 - 22.4), and injuries resulted in hospitalization was 16.7 (14.2 - 19.6) per 1000 population. The annual incidence of fatal injuries was 33 (7-96) per 100,000 Population. In children aged 15 or less, the annual incidence of all injuries was 137.2 (120.0 - 155.9), significant injuries needing any medical care was 64.2 (52.2 - 78.0), fractures was 21.8 (15.0 - 30.7), and injuries resulted in hospitalization was 6.8 (3.3 - 12.5) per 1000 population. The mean out of pocket medical expense for injuries was 19.9 USD.

**Conclusion:**

This population based study showed that the real incidence of non-fatal injuries in the capital of Iran is more than the formal hospital-based estimates. These injuries impose non trivial medical and indirect cost on the community. The out of pocket medical expense of non-fatal injuries to Tehran population is estimated as 27 million USD per year. Effective strategies should be considered to minimize these injuries and decrease the great financial burden to public and the health system.

## Background

Injuries are the cause of nine percent of all deaths and 16% of the disability adjusted life years (DALY) lost worldwide [1]. It is expected that the impact of injuries on population health increases in the future [2]. Fatal injuries are at the top of the injury pyramid; however, non-fatal injuries are quite common and impose huge medical expenses on the population. For every fatal injury in the UK, 45 hospital episodes, 630 physician consultations, and 5000-6000 minor injuries are recorded [3]. The majority of studies on injuries in developing countries depend on hospital data. Admission to the hospital due to injury depends on the severity of injury, access to hospital services, the health system configuration, and some health economic factors [4]. Therefore, use of hospitalization data will underestimate the incidence of non-fatal injuries.

Although most of the traumatic deaths occur in low and middle income countries [5], these countries generally lack efficient surveillance systems to monitor the injury incidence [6].

There are some studies conducted in Iran to assess the trauma epidemiology in patients who referred to hospitals [6,7], little if any, have evaluated the incidence of injuries irrespective of visiting hospitals. We conducted a household survey to estimate the annual incidence and out of pocket medical expenses of all injuries in urban population of Tehran (the capital city of Iran) with a population of 7.9 million. The population based approach of this study prevents under estimation and depicts the epidemiology of non-fatal injuries in the capital city of Iran, to gain more attention from policy makers.

## Methods

Using the cluster random sampling approach, we arranged a household survey one month after the end of the Persian calendar year at April 2008. Postal address registry of greater Tehran was considered as the sampling frame; ninety seven residential location addresses were randomly selected out of the 4084203 in the registry. Residential locations were considered as buildings that were registered as residence rather than office etc.

Each address served as a starting point of the cluster. Interviewers referred to the starting points and moved counterclockwise to capture 25 residential locations, leaving three addresses in between. In case of complexes with several apartments, the first apartment was included and the next three were skipped and the forth apartment was included. Interviewers spent a full day in every cluster. If an adult person was not found at the selected residential location, the interviewers referred to that location two more times on the same day, and then continued to capture the 25 households in the same cluster by substituting non-responders if necessary. For substitution, they continued the same approach as described above.

At selected residential locations, interviewers asked one adult person (preferably one of the parents) who was at home and accepted to take part in the study to report all injuries which have occurred during the past Persian calendar year for all household members (defined as people living in the same residence), as well as the care provided and the amount spent as the out of pocket medical expenses. Informants were provided with explanation on study objectives and the exact time period of interest. They were asked to report the type of injury, place of occurrence, the activity, cause of accidents resulting in injuries, the amount of out of pocket medical expenses for injury, and whether they referred to hospital. The following injuries were considered as needing medical care: fractures, dislocations, amputations, meniscal tear, tendon injuries, concussion, ocular injuries, internal bleeding, respiratory tract irritation due to inhalation of an irritating gas and poisonings. Open wounds that needed suture or a medical treatment to stop bleeding, grade two and three burns, sprains and crushing injuries that resulted in disability for two days or more were also considered as needing medical care.

The out of pocket medical expenses included any expenditure on diagnosis and treatment of the injury that patients had to pay out of their own pocket and were not covered by any insurance. This included health visits, medications, radiology, laboratory, service fees, hospitalization, medical equipment and rehabilitation.

Furthermore, they were asked to list their household members and their demographic characteristics. Detected injuries and mechanisms were later coded according to International Classification of Diseases and related health problems tenth version (ICD-10), by a single trained physician. The reported injuries were classified into the following categories: all injuries, significant injuries (defined as injuries needing medical care), and injuries necessitating hospital admission. The incidence rates of injuries in age-sex categories were calculated. The household members aged ≤ 15 were considered children and their data are presented separately. The mean out of pocket medical expenses was calculated per injured persons.

The mean housing price in the residential area of households was collected from the official report of Iranian Ministry of Housing and Urban Development [8]. The annual mean exchange rate of United States Dollar (USD) with Iranian currency (Rials) was obtained from the Central Bank of Iran http://www.cbi.ir/exratesadv/exratesadv_fa.aspx.

The following formula was used to estimate the minimum needed sample size with the precision of 1% while α was set to 5%: 

The annual incidence of all injuries (p) was assumed to be about 25 per 1000 person-years (12). Then, we multiplied the result by 1.3 to deal with the design effect.

Data were analyzed using STATA 8 SE and clustered structure of sampling was taken into account during the analysis. Injury incidences (cumulative) are presented as incidence per 1000 populations (± 95% Confidence interval); confidence intervals were calculated assuming Poisson distribution. Continuous variables were compared using student's t test. Informed consent was obtained from all the participants, and the study was approved by the Review Board of Sina Trauma and Surgery Research Center.

## Results

This study included 2,450 households residing in Tehran during 2007-8. We approached 2871 households; 324 were not found at home, 94 declined to participate and 2450 were included. The sample consisted of 4,707 male and 4,393 female household members (Table 1), including 1465 children.

**Table 1:** Annual incidence (per 1000) of injuries in age-sex groups of study population, all injuries

Age (years)	Gender										Weighted overall Incidence Rate (per 1000) (95% CI)	
			
	Male					Female						
				
	Study population	Injuries (N)		Incidence		Study population	Injuries (N)		Incidence			
				
		All	Significant	All	Significant		All	Significant	All	Significant	All	Significant
< = 5	211	22	11	104.3	52.1	184	13	3	71.7	16.3	88.6 (62.5-121.1)	35.4 (19.5-58.8)
6-15	547	101	59	184.6^B^	107.9^A^	523	65	21	124.3 ^B^	40.2^A^	155.1 (134.0-178.2)	74.8 (59.7-92.2)
16-25	1187	171	126	144.1	106.1 ^A^	991	158	33	159.4	33.3 ^A^	151.1 (136.3-166.8)	73.0 (62.4-84.7)
26-35	808	112	102	138.6 ^A^	126.2 ^A^	785	274	32	349.0 ^A^	40.8 ^A^	242.3 (221.4-264.1)	84.1 (71.0-98.8)
36-45	647	73	57	112.8 ^A^	88.1 ^A^	740	255	29	344.6 ^A^	39.2 ^A^	236.5 (211.6-263.5)	62.0 (49.6-76.6)
46-55	586	56	37	95.6 ^A^	63.1	620	197	27	317.7 ^A^	43.5	209.8 (184.7-237.3)	53.1 (40.9-67.8)
56-65	394	37	25	93.9 ^A^	63.5	359	99	19	275.8 ^A^	52.9	180.6 (151.5-213.7)	58.4 (42.5-78.4)
66-75	243	33	20	135.8	82.3	147	30	11	204.1	74.8	161.5 (124.1-206.7)	79.5 (54.0-112.8)
76+	84	9	3	107.1 ^B^	35.7 ^A^	45	12	11	266.7 ^B^	244.4 ^A^	162.8 (100.8-248.8)	108.5 (59.3-182.1)

Total	4707	614	440	130.4 ^A^	93.5 ^A^	4393	1103	186	251.0 ^A^	42.3 ^A^	188.7 (180.7-196.9)	68.8 (63.7-74.2)

^A^: There is statistically significant difference in the incidence this type of injury between men and women; P < 0.001

^B^: There is statistically significant difference in the incidence this type of injury between men and women; P < 0.01

The informants reported a total of 1937 injuries during the past Persian year, of which 626 injuries (32.3%) needed medical care. The reported number of all injuries among children was 201, among them 94 (46.8%) needed medical care. Two hundred and twenty persons had experienced more than one injury in accidents; therefore, the number of injured persons was 1717 (18.9%). One hundred and fifty two (8.9%) injured persons reported hospital admission for the injury.

**Figure 1:**
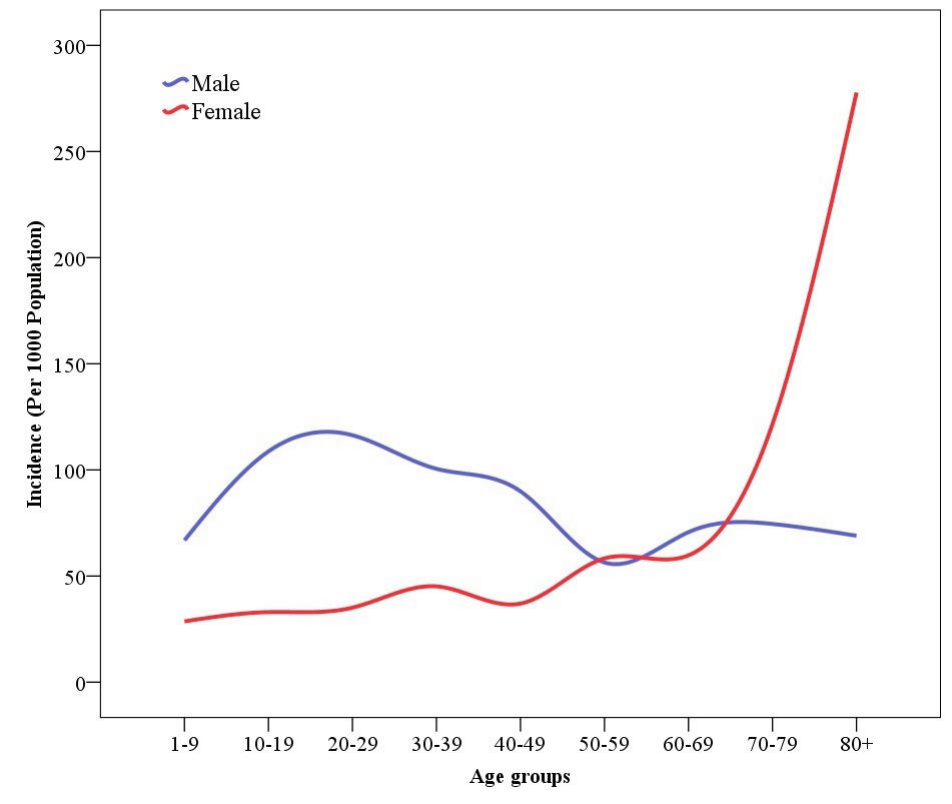
**Annual incidence of significant injuries according to gender and age groups**.

While more injuries were reported for women of age 16 or more (Table 1), significant injuries that needed medical care were more common in men (Figure 1) up to the age of 70. In children, the incidence of both significant and all injuries were higher among males (P < 0.001).

Home was the place that most of the injurious accidents had happened (Table 2). Significant injuries needing medical care mostly happened in the streets and highways (Table 2). The same pattern was seen in children.

**Table 2:** Place of occurrence of accident ^‡^

Place of occurrence	All accidents N (%)	Accidents resulted in significant injury§ N (%)					
		
		Age groups		Common causes of accidents			
		
		All ages	Age ≤ 15	Transport accidents	Falls	Inanimate mechanical force	Contact with heat and hot substances
Home	1059 (61.7)	140 (22.4)	23 (24.5)	0 (0.0)	55 (25.7)	32 (35.6)	46 (76.7)
Street and highway	365 (21.3)	296 (47.3)	33 (35.1)	202 (99.5)	58 (27.1)	6 (6.7)	7 (11.7)
Sports and athletics area	86 (5.0)	65 (10.4)	23 (24.5)	0 (0.0)	52 (24.3)	5 (5.6)	0 (0.0)
Trade and service area	65 (3.8)	49 (7.8)	2 (2.1)	0 (0.0)	0 (0.0)	33 (36.7)	2 (3.3)
School, other institution and public administrative area	20 (1.2)	9 (1.4)	6 (6.4)	0 (0.0)	8 (3.7)	0 (0.0)	0 (0.0)
Industrial and construction area	18 (1.0)	15 (2.4)	0 (0)	0 (0.0)	0 (0.0)	5 (5.6)	4 (6.7)
Farm	1 (0.1)	1(0.2)	0 (0)	0 (0.0)	0 (0.0)	1 (1.1)	0 (0.0)
Other specified places	4 (0.2)	4 (0.6)	1 (1.1)	0 (0.0)	3 (1.4)	1 (1.1)	1 (1.7)
Unspecific places	99 (5.7)	47 (7.5)	6 (6.4)	1 (0.5)	38 (17.8)	7 (7.8)	0 (0.0)

Total ‡	1717 (100.0)	626 (100.0)	94 (100.0)	203 (100.0)	214 (100.0)	90 (100.0)	60 (100.0)

‡ Accident are appeared only once in this table even if it was resulted in more than one injury

§ Significant injury: Injuries that needed any medical care

Significant injuries were most likely to happen while working to earn an income (Table 3) while all injuries were most likely to happen while being engaged in other types of work such as cooking, cleaning, household maintenance, and caring for children (Table 3).

**Table 3:** Activities during which the accidents^‡ ^occurred

Activity	All accidents N (%)	Accidents resulted in significant injury§ N (%)				
		
		Total	Transport accidents*	Falls	Inanimate mechanical force	Contact with heat and hot substances
While engaged in Other types of work	1009 (58.8)	131 (20.9)	0 (0.0)	53 (24.8)	28 (31.1)	46 (76.7)
While working for income	404 (23.5)	338 (54.0)	189 (93.1)	63 (29.4)	54 (60.0)	14 (23.3)
While engaged in Leisure activity	130 (7.6)	58 (9.3)	12 (5.9)	42 (19.6)	2 (2.2)	0 (0.0)
While engaged in Sports activity	89 (5.2)	70 (11.2)	2 (1.0)	54 (25.2)	6 (6.7)	0 (0.0)
While resting, sleeping, eating or other vital activities	4 (.2)	0 (0.0)	0 (0.0)	0 (0.0)	0 (0.0)	0 (0.0)
While engaged in other specified activities	30 (1.7)	22 (3.5)	0 (0.0)	2 (0.9)	0 (0.0)	0 (0.0)
During unspecified activity	51 (3.0)	7 (1.1)	0 (0.0)	0 (0.0)	0 (0.0)	0 (0.0)

Total‡	1717 (100.0)	626 (100.0)	203 (100.0)*	214 (100.0)	90 (100.0)	60 (100.0)

‡ Accident are appeared only once in this table even if it was resulted in more than one injury

§ Significant injury: Injury that needed any medical care

* The distribution of RTCs was as follows: 96 (47.3%) were motorcycle rider, 82 (40.4%) were pedestrian and 25 (12.3) were car occupant.

Inanimate mechanical forces such as falling of heavy objects, contact with a sharp glass, knife, powered and non-powered hand tools (scissors, can-openers, handsaw, needle, etc.), and discharge of fireworks were the most common causes of occurrence for all injuries (Table 4). Falls followed by transport crashes were the most common causes of significant injuries (Table 4) in all ages including children. The distribution of RTCs that resulted in significant injuries was as follows: 96 (47.3%) were motorcycle riders, 82 (40.4%) pedestrians and 25 (12.3) were car occupants.

**Table 4:** Causes of accidents^‡ ^resulting in injuries

Cause	All injuries N (%)	Out of pocket medical expenses (USD) ** Mean ± SD	Significant injuries^§^					
			
			All ages					Age ≤ 15 N(%)
				
			Male		Female		Total N(%)	
					
			N	Incidence*	N	Incidence*		
Inanimate mechanical force	629 (36.6)	19.4 ± 7.2	67	14.2 (11.0-18.0)	23	5.2 (3.3-7.8)	90 (14.4)	7 (7.4)
Falls	403 (23.5)	22.1 ± 10.3	126	26.8 (22.3-31.8)	88	20.0 (16.1-24.6)	214 (34.2)	54 (57.4)
Contact with heat and hot substances	372 (21.7)	19.4 ± 7.3	24	5.1 (3.3-42.6)	36	8.2 (5.8-11.3)	60 (9.6)	7 (7.4)
Transport accidents	188 (10.9)	18.7 ± 7.9	174	37.0 (31.8-42.8)	26	5.9 (3.9-8.7)	203 (32.4)	21 (22.3)
Others	50 (2.9)	18.9 ± 9.1	5	1.1 (0.3-2.8)	1	0.2 (0.0-1.3)	3 (0.5)	0 (0.0)
Assault	44 (2.6)	18.8 ± 8.5	34	7.2 (5.0-10.1)	1	0.2(0.0-1.3)	35 (5.6)	3 (3.2)
Exposure to smoke, fire, flame	13 (.8)	18.9 ± 4.7	3	0.6 (0.1-1.9)	2	0.5 (0.1-1.6)	5 (.8)	0 (0.0)
Overexertion,...	8 (.5)	19.3 ± 6.7	5	1.1 (0.3-2.8)	3	0.7 (0.1-2.0)	8 (1.3)	0 (0.0)
Electric current	6 (.3)	17.4 ± 5.9	2	0.4 (0.1-1.5)	2	0.5 (0.1-1.6)	4 (.6)	0 (0.0)
Animate mechanical force	2 (.1)	14.1 ± 2.0	0	0.0 (0.0-0.8)	2	0.5 (0.1-1.6)	2 (.3)	1 (1.1)
Poisoning, accidental	2 (.1)	31.7 ± 26.7	0	0.0 (0.0-0.8)	2	0.5 (0.1-1.6)	2 (.3)	1 (1.1)

Total‡	1717 (100.0)	19.9 ± 8.3	440	93.5 (85.3-102.2)	186	42.3 (36.6-48.7)	626 (100.0)	94 (100.0)

‡ Accident are appeared only once in this table even if it was resulted in more than one injury

§ Significant injury: Injury that needed any medical care

* Incidence per 1000 person-year

** The mean exchange rate for 1 USD was 9285 Rials during the study period

Fractures comprised 9.1% of all injuries and 27.7% of significant injuries. Fall was the most common cause for fractures (Table 5). Those men who reported a fracture were younger than women (Table 5, P < 0.001). Thirty two cases of fracture had happened in children; this constituted 34.0% of significant injuries in this age group. The causes of fracture in children were fall (87.5%), transport accident (9.4%) and animate mechanical force (3.1%).

**Table 5:** Cause of fractures

Cause	N (%)	Age	
		
		Male	Female
		
		Mean ± SD	Mean ± SD
Falls	109 (61.9)	28.2 ± 20.7	44.6 ± 24.4
Transport accidents	53 (30.1)	39.9 ± 19.9	43.9 ± 16.2
Inanimate mechanical force	6 (3.4)	29.7 ± 5.5	55.0 ± 26.9
Animate mechanical force	1 (0.6)	--	15.0
Overexertion, travel and privation	1 (0.6)	30.0	--
Assault	6 (3.4)	27.2 ± 6.8	--

Total	176 (100.0)	32.6 ± 20.2	44.5 ± 23.2

**Figure 2:**
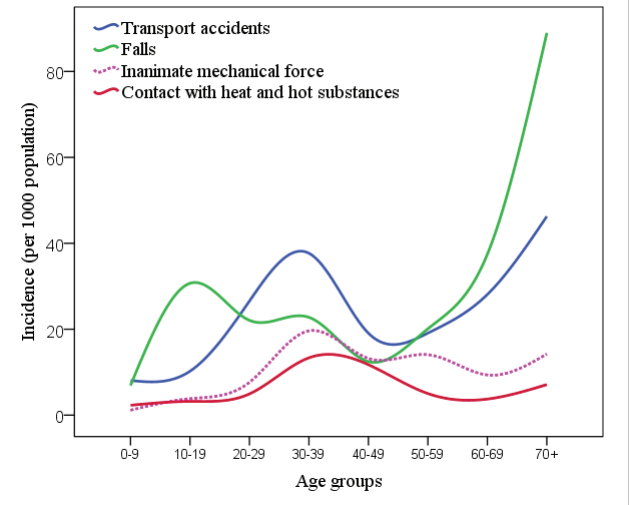
**Annual incidence of injuries due to transport accidents, falls, inanimate mechanical force and contact with heat and hot substances, according to age groups**.

Falls and transport accidents were the most common causes of reported significant injuries (table 4). Falls were most common in the young age groups with another peak in elderly while transport accidents were most common in middle age groups with another peak in elderly that was less prominent than falls (Figure 2). While the incidence of significant injuries due to falls was similar in both genders (table 4), transport accidents were six times more common in significant injuries reported in men compared to women (table 4).

Contact with heat and hot substances and also inanimate mechanical force were the next two common causes of reported significant injuries (table 4), and they both were most common in the middle age groups (Figure 2). While contact with heat and hot substance was more common in women, injured men had experienced inanimate mechanical force more than women (table 4).

The annual incidence of all injuries was 188.7 (180.7-196.9), significant injuries needing any medical care was 68.8 (63.7-74.2), fractures was 19.3 (16.6 - 22.4), and injuries resulted in hospitalization was 16.7 (14.2 - 19.6) per 1000 population. The annual incidence of fatal injuries was 33 (7-96) per 100,000 Population. In children aged 15 or less, the annual incidence of all injuries was 137.2 (120.0 - 155.9), significant injuries needing any medical care was 64.2 (52.2 - 78.0), fractures was 21.8 (15.0 - 30.7), and injuries resulted in hospitalization was 6.8 (3.3 - 12.5) per 1000 population.

The mean out of pocket medical expense for injuries was 19.9 USD. The highest out of pocket medical expense was for traumatic amputation while the least amount was for superficial injuries. The study population had spent a sum of 31,216.1 USD for out of pocket medical costs of non-fatal injuries during the study period.

The mean housing price in the residential area of households who reported an injury was 2107.9 ± 904.3 USD and for households who did not report an injury was 2197.3 ± 1000.4 USD (P = 0.02).

## Discussion

The aim of this study was to estimate the incidence and out of pocket medical expenses of all injuries in the urban population of Tehran -the capital of Iran- with a population of 7.9 million.

To avoid under-estimation of injury incidence, we used a household survey. Hospital based data could be misleading when they are used to estimate non-fatal injuries [9] as not all injuries are admitted to hospitals. Admission to hospital is influenced by severity of injury [10] as well as the health system structure, the public's access to services, and presence of alternative solutions (private sector, non-physician clinicians, etc).

We referred to the study subjects at the beginning of the new Persian year and asked them to report all injuries that happened during the last calendar period. This approach may minimize the probability of recall bias, because the study period is well defined and the starting point and end point of the study period are anchored in the memory of informants. However, this does not preclude possibility of recall bias, especially for injuries that had happened earlier in the study period. Unfortunately, we were not able to compare the injury incidence during the last three months with the similar time intervals earlier in the study period because we did not collect the date of occurrence of reported injuries. Therefore, we cannot assess the extent of potential recall bias and this is a major limitation. The majority of household members had close familial relationships; therefore, it was unlikely that the responding adult person was unaware of injuries of other household/family members, especially for significant injuries. Again, the possibility of recall bias for minor injuries occurred earlier in the study period could not be ruled out. However, this study showed that the real incidence of injury in the capital of Iran is more than the formal estimates that are hospital based. The recall bias will cause some residual underestimation that is not addressed in our study but it does not invalidate the whole message as the first population based study in the Capital of Iran.

The majority of injuries detected in this survey were minor injuries. Safety is defined as a state in which hazards and conditions leading to harm are controlled in order to preserve health [11]. An out of control hazard could result in minor injury -in most cases- or in some cases may proceed to severe injuries. It is important to keep control of hazards to prevent injuries either minor or severe. Occurrence of a minor injury indicates that the control on hazards is lost and this points out the importance of monitoring all injuries (including minor) incidences and their trend in the community.

The crude annual incidence of all non-fatal injuries was 188.7 per 1000 population. This is less than estimates of a similar study from Sri lanka that was 246 per 1000 population [12]. Our study was performed in an urban area. The incidence of injuries in urban areas is less than rural areas [13,14]. This may explain the lower estimate of non-fatal injury in our study compared to Sri lanka. The incidence of injuries resulting in hospitalization was 16.7 per 1000 population. This is similar to another population based study from Iran that reported a 10 per 1000 population hospitalization due to injury [15]. Their study were located in a less developed region of Iran; the slightly higher hospitalization rate in our study may be due to better access of capital inhabitants to referral health centers as well as health demands induced by private centers in the capital.

There was a peak in occurrence of injuries due to RTC, inanimate mechanical force and contact with heat and hot substances in middle ages. A peak in occurrence of fall injuries in the second decade of life may be due to careless manner of this age group while playing in unprotected areas. The other peak in elderly may be due to imbalance and osteoarthritis. Toll of fall injuries in this age group is increased because of osteoporosis. Environmental modifications to protect children and elderly from fall, should be considered by municipality authorities. Elderly people were found to be at increased risk of RTIs, reflected by the second peak in TRI incidence in this age group. This may be explained by impaired balance and also by non-effective separation of pedestrians from motorized vehicles. Specifically, motorcycles may even drive in pavements and endanger elderly people who are not able to react promptly to protect themselves. Law enforcement should be considered to effectively separate motor vehicles from pedestrians.

We considered the mean housing price in the residential area of informants as an indicator of their socio-economic status of the households and found an inverse association with injury incidence. There are reports indicating an association between lower socio-economic status and fatal injury; however, the evidence of socioeconomic inequalities in non-fatal injury rates has not been wholly consistent [16]. Zarzaur et al. reported that neighborhood socioeconomic status was inversely related to crude injury rates for all mechanisms [17]. Low housing prices in a specific residential area in Tehran is the result of overcrowding, poor access to urban servitudes, unsatisfactory urban design and sub-standard condition of buildings. People residing in areas with lower housing prices, have less resource to improve the safety status of their houses such as installing handrails or fences to prevent fall injury. Besides, people residing in low-priced areas are more likely to have the type of jobs that expose them to occupational injuries. It seems that public education on injury prevention needs to be reinforced in residential areas of lower socio-economical classes.

Significant injuries were more common in men. This is consistent with reports from other countries [12] and with a report of DALY of diseases and injuries in Iran in the year of 2003 [18] that was prepared based on hospital data. The reason may be occupational exposure, risk taking behaviors and more likelihood of men to involve in violence. The higher rates for road traffic crashes and assault in men compared to women have been reported in other countries [16].

We also noted higher incidence of significant injuries in women older than 70 compared to men. The number of participants in our sample was small in older age groups; this may result in unstable estimate of incidence. However, there are reports of higher fall injuries and also fall induced fracture in elder women compared to men [19].

Most of significant injuries in our study had occurred while working for income. This reminds the need to improve the safety of working places especially in case of small workshops. There are obligatory safety standards for large industrial units in Iran but small workshops fail to meet such standards. Moreover, there are many seasonal workers that are hired to work in private building activities and their employers do not follow any safety rule. At the same time, only a small proportion of seasonal workers have insurance coverage.

While significant injuries mostly happened in streets and highways, home was the place that most of injuries (regardless of severity) had happened. Gorman et al. reported that home, work/school; street/public locations and sport leisure activity related places were respectively the most common places of occurrence of injury in UK [20]. This is in accordance with our findings (Table 2). Home is known as a setting in which un-intentional injuries occur [21]. Safety promotion in houses is deemed to result in a considerable decrease in unintentional injury among the public [21]. Home safety education, especially with the provision of safety equipment is reported to increase the safety practices [22]. Mandatory safety standards for houses such as safe windows, fire alarms, etc. may be considered by civil authorities. Special consideration is needed for passive strategies to prevent fall, as it was the most common cause of significant injuries. Households need to be aware of the risk of inanimate mechanical forces in the houses. Technical advice on how to prevent heavy object from dropping at home and how to keep sharp objects at home should be provided to all households in a systematic manner.

Falls were the most common cause of fractures and other significant injuries. Surveys from Vietnam, Tanzania, Pakistan, India, china Ghana, Nicaragua and Nigeria have reported falls as the leading cause of non-fatal injuries [12,14]. The incidence of fracture was 19.3 per 1000 population. It imposes non trivial medical and indirect cost on the community. The medical out of pocket expense for fractures is estimated about 12 million USD for Tehran population. It is necessary to prevent falls to decrease this expense.

The injury pyramid in our study population was 1/50/646 (Death/Hospital admission/All injuries). This is similar to injury pyramid in the general population in UK that is reported as 1/45/630 (deaths/hospitalizations/any medical treatment) [16].

The injury pyramid is reported as 1/10/178 for USA and 1/45/267 for Australia (deaths/hospitalizations/Accident & Emergency attendance) [16] and 1/25/354 for a Nicaraguan community (deaths/moderate to severe/and minor injuries) [23]. Although the injury pyramid in our study was similar to UK, this ratio may not be stable enough for comparison with other countries due to small number of fatal injury in our study population. Moreover, the comparison of injury pyramid between different countries may not be straightforward as it takes effect from numerous factors including the health system utilization.

Falls followed by transport related injuries were the leading causes of significant injuries. This was similar to reports from UK indicating that RTC and falls are the leading causes of fatal and non-fatal injuries in children and the general population [16]. The same pattern is reported in China [14].

The study population had spent a sum of 31216.1 USD for out of pocket medical costs of all injuries during the studied year. Projecting this to the Tehran population indicate that non-fatal injuries impose about 27 million USD out of pocket medical cost to Tehran population annually. As a point of reference, the mean household income for an Iranian urban household in 2006 was equivalent to $7,055 U.S. [24]. The Health Care system in urban areas in Iran is a combination of both public and private systems. Patients are allowed to choose their healthcare provider. The cost of medical service in the private sector is higher than the public. Employed people and their family members up to the age of 18, have medical insurance; however, this is not mandatory for self-employed people. The out of pocket medical expenses for insured people are supposed to be 20% for in-hospital care. In reality, however, the insurance companies pay a fraction of the total medical expenditure. Therefore, the out of pocket medical expenses may come out to be high, especially in the private sector.

However our study has some limitations; a proxy informant provided information on injuries for all household members. It is possible that the respondents under-reported the minor injuries happened to other household members; however, this is less likely in case of significant injuries that needed medical care.

Although the time period was clearly defined for participants, recent injuries are more likely to be recalled compared to earlier one, especially in case of minor injuries of other household members and we were not able to evaluate the potential recall bias as we did not record the exact time of occurrence of injuries. This issue should be addressed in future studies.

It is likely that this study underestimated the incidence of injuries associated with a social stigma i.e. domestic violence, sexual assault or suicide. We were not able to explore the extent of probable under reports of above mentioned injuries. However, we believe that the estimates of this study are less prone to under estimation of the non-fatal injury incidences compared to service based studies.

## Conclusion

This study is the first to describe the incidence and out of pocket medical expenses of all injuries in a major city in Iran using a population based survey. According to our study the real incidence of non-fatal injuries is more than the formal estimates that are hospital based. These injuries impose non trivial medical and indirect cost on the community. Underestimation of the injury problem and consequently failure to use effective strategies to control it continues to impose a great financial burden to public and the health system.

## Abbreviations

DALY: disability adjusted life years; ICD-10: International Classification of Diseases and related health problems tenth version; USD: United States Dollar.

## Competing interests

The authors declare that they have no competing interests.

## Authors' contributions

SS designed the study, managed data collection, analyzed data and composed manuscript draft and edited final manuscript. MM performed the literature search and contributed to manuscript draft. MS contributed to data collection, analysis and drafting and also edited the draft. All three authors read and approved the final manuscript.

## Pre-publication history

The pre-publication history for this paper can be accessed here:

http://www.biomedcentral.com/1471-2458/11/608/prepub
